# A comparison of the Oxford shoulder score and shoulder pain and disability index: factor structure in the context of a large randomized controlled trial

**DOI:** 10.2147/PROM.S115488

**Published:** 2016-11-21

**Authors:** Jill Dawson, Kristina K Harris, Helen Doll, Ray Fitzpatrick, Andrew Carr

**Affiliations:** 1 Nuffield Department of Population Health; 2 Nuffield Department of Orthopaedics, Rheumatology and Musculoskeletal Sciences, University of Oxford; 3 ICON Patient Reported Outcomes, Oxford, UK

**Keywords:** shoulder, Oxford shoulder score, shoulder pain and disability index, patient-reported outcome measures, factor analysis

## Abstract

**Background:**

To explore and compare the factor structure of the 12-item Oxford shoulder score (OSS) and 13-item shoulder pain and disability index (SPADI).

**Methods:**

Exploratory factor analysis (EFA) and confirmatory factor analysis (CFA) of data from 660 patients attending 46 hospitals in the UK. Complete OSS and SPADI data were available for 648 (98.2%) and 628 (95.2%) participants, respectively.

**Results:**

For both instruments, either one or two factors were indicated, depending on the extraction method. On EFA, most OSS items loaded saliently on either of two “Pain” (4 items) and “Function” (8 items) factors, although some items cross-loaded. Cronbach’s alphas were 0.75, 0.90, and 0.91 for “Pain” and “Function” subscales, and all 12 OSS items, respectively. CFA suggested marginally better fit for two factors, with neither one- nor two-factor models rejected. EFA indicated two factors for the SPADI, with three of the eight “Disability” items contributing to an 8-item “Pain factor”, with 2 items within the 5-item “Disability factor” cross-loading. Cronbach’s alpha was 0.87 and 0.93 for the original 5- and 8-item pain and disability scales; 0.94 for all 13 SPADI items, respectively. CFA suggested marginally better fit for the two-factor (original conceptualization) model of the SPADI, with neither one- nor two-factor models rejected.

**Conclusion:**

EFA and CFA demonstrated that, in addition to single summary scales usage, separate information on pain and self-reported disability/function can be extracted in a meaningful way, as subscales, from both the OSS and the SPADI. This information can help researchers in choosing primary study endpoints appropriately.

## Introduction

Patient-reported outcome measures (PROMs) are standardized questionnaires (instruments) designed to measure particular constructs of patients’ health status, from their perspective, in defined populations. Familiarity with psychometric methodology (used to develop and validate PROMs) has increased, and recommendations have multiplied and become more stringent in guiding health status measures’ development and evaluation (eg, Streiner and Norman;^1^ FDA guidelines^2^). The availability of large datasets representing particular patient characteristics, diagnostic or treatment groups, has also facilitated further investigation of measurement properties (eg, reliability, validity, and responsiveness) of instruments used to assess health care outcomes. This is important, because the measurement properties of individual PROMs are not immutable properties of the instruments alone, but relate to the population, condition, or treatment studied.

Factor analysis (FA) embodies a number of statistical methods applicable to the development and validation of PROMs, where the purpose is to identify or confirm latent factor solutions that can explain the pattern of correlations or covariances between the observed variables (questionnaire items).^3^

This paper aims to explore the underlying structure of two widely used PROMs: the Oxford shoulder score (OSS)^4^,^5^ and shoulder pain and disability index (SPADI),^6^ both developed in the 1990s to assess shoulder pain and function. Exploration of the factor structure of the original English version of the OSS has not previously been reported, while exploratory factor analysis (EFA) results (without rotation, using a small sample) were reported for the SPADI in its developmental study^6^ and later, from a large population-based study.^7^

Secondary data, used for these analyses, were collected within a large-scale surgical trial, the UKUFF rotator cuff randomized controlled trial (RCT),^8^,^9^ with patients recruited to be representative of the target patient population. By assessing whether, within this context, pain and functioning/disability as measured by the OSS and SPADI can be distinguished in a meaningful way, future researchers will be able to identify the most appropriate study endpoints for future clinical trials.

## Materials and methods

### Secondary data analysis

The study sample included 660 patients attending 46 hospitals across the UK, who from November 2007 until February 2012, were recruited/randomized as part of the UKUFF trial.^8^,^9^ Eligibility criteria: patients aged ≥50 years, able to give informed consent, symptoms of a degenerative full-thickness rotator cuff tear, deemed suitable for rotator cuff repair surgery, where the surgeon was uncertain which surgical procedure was better. Patients were randomized to receive either open rotator cuff repair (n=219), arthroscopic rotator cuff repair (n=227), or “rest then exercise” (n=214). For this study, only baseline data were used in the analyses. Full details of trial methods, sample characteristics, and results are published elsewhere.^8^,^9^ All subjects gave their informed written consent to participate. The study was approved by the UK National Health Service (NHS) Research Ethics Committees (RECs) (UKUFF REC reference number 10/H0402/24).

### Outcome measures

The trial included two shoulder-specific PROMs:

The OSS was developed using in-depth interviews with patients attending shoulder surgery outpatient clinics from which were identified salient themes for informed questionnaire item content. Items were pretested and amended/reduced in response to patients’ feedback.^4^ Rigorous assessment of reliability, validity, and responsiveness occurred within prospective studies employing classical psychometric methods.^4^,^10^ The OSS was designed to be used as a composite scale, reflecting patients’ perceptions of shoulder pain and functional impairment frequently described as being inextricably linked. The measure was primarily developed for the assessment of outcomes of shoulder surgery (excluding shoulder stabilization, for which there is a separate, specific PROM – the Oxford shoulder instability score^11^) in randomized trials. Comprising 12 items, each referring to the past 4 weeks, the OSS has been shown to be highly responsive in the surgical context.^4^,^10^,^12^ Each item offers 5 ordinal response options, originally scored from 1 to 5 (5= most severe), then summed to produce a summary score range of 12–60. The recommended method of scoring subsequently changed^5^ to score each item from 0 to 4, with 4 representing the best (ie, the opposite direction from the original method). The 12 summed items produce an overall score from 0 to 48 (48= best outcome).

The SPADI is also a self-administered questionnaire, developed by a panel of rheumatologists and a physiotherapist, to measure shoulder pain and disability in an outpatient setting.^6^ Evidence has been reported supporting the reliability, validity, and responsiveness of the SPADI.^13^ It was conceptualized to measure shoulder pain and disability separately, using two subscales, with the option of producing one overall score, and containing 13 items (5 assess shoulder pain, beginning: “How severe is your pain[…]”; while 8 assess disability, beginning: “How much difficulty do you have […]”), all with reference to the last week. The original version scored items on a visual analog scale (VAS). A second version, used in this trial, replaced the VAS with 0–10 numerical rating scales.^13^,^14^ Item responses within each subscale are summed and transformed to a score out of 100. A mean is taken of the two subscales to give a total score out of 100 (100= greatest impairment/disability, ie, the opposite direction from the OSS).

## Analysis

EFA and confirmatory factor analysis (CFA), using PASW Statistics 20 (Copyright 2015, SPSS Inc., SPSS [Hong Kong] Ltd., Hong Kong), FACTOR,^15^ and LISREL 9.10 structural equation modeling software (Copyright 2005–2014, Scientific Software International, Inc., Skokie, IL, USA), analyzed OSS and SPADI responses using complete data, ie, where responses to all OSS or SPADI items were received from a patient.

FA is a procedure that is widely recommended and used in the construction and validation of PROMs.^1^,^3^,^16^,^17^ By analyzing the pattern of correlations or covariances between the observed variables/items, the main goal of the EFA procedure is to explain the observed variables (for PROMs, items on a scale) by a smaller number of latent variables (factors).^16^,^18^ By contrast, CFA tests the fit of a priori hypothesized structures of an instrument statistically. Usually, several competing models that are based on theory and/or empirical research are tested for goodness of fit. Both EFA and CFA assume normally distributed data when using Pearson-product moment correlations. Where measures have categorical (ordinal) responses, analyses should instead be based on the matrix of polychoric correlations, which is robust to underlying non-normality.^19^,^20^

### EFA

Data suitability for EFA was assessed using the Kaiser–Meyer–Olkin measure of sampling adequacy and Bartlett’s test of sphericity. Criteria for suitability are Kaiser–Meyer–Olkin >0.8 and a *P*-value for Bartlett’s *x*^2^ of <0.01.^18^ As the goal of EFA was to identify the number of factors that the measure was assessing, principal axis factoring was chosen as the extraction method.^16^,^21^ The decision on the number of factors to extract was assessed using several methods: Kaiser over 1 rule,^22^ scree test,^23^ Velicer’s minimum average partial (MAP) test,^24^ and Horn’s parallel analysis (PA).^25^

Factors were explored using oblique (Promax) rotation method (which allows correlation between extracted factors). An item was considered to load on a factor if it had a pattern matrix loading >0.3.^26^ The internal consistency of any identified, and original, subscales was tested with Cronbach’s alpha.^27^ Alpha values in the range of 0.80–0.90 are considered optimal, with a minimum alpha of 0.70 being necessary to claim internal consistency.^26^,^28^

CFA was conducted to test the fit of different hypothesized factor models for the OSS and SPADI.

### OSS

**Model 1** hypothesized that all 12 items characterize a single underlying factor representing the conceptual basis of the OSS.^8^ The acceptability of this model was further confirmed by evidence of its high internal consistency, based on Cronbach’s alpha, and on the basis of the number of extracted factors in this study using some of the most commonly recommended methods, namely Parallel analysis^29^ and Velicer’s MAP test.^24^
**Model 2** tested two first-order correlated factors, as indicated by other commonly recommended methods within the EFA: scree test,^23^ K-over-1 rule^2^,^2^ and Horn’s PA.^25^ Based on these EFA results, items 1, 8, and 12 were assigned to the “Pain” factor and other items (including cross-loading items 2 and 11) to the “Function” factor.
**Model 3** was based on the same principles as Model 2, except that the cross-loading item 11 was assigned to the “Pain” factor (together with items 1, 8, and 12).
**Model 4** was based on the same principles as Models 2 and 3, except that cross-loading items 2 and 11 were here assigned to the “Pain” factor.

### SPADI

**Model 1** hypothesized that all items characterize a single underlying factor. This model was tested as the SPADI may also be used in this way.^6^
**Model 2** tested two first-order correlated factors using the two-factor model corresponding to the conceptual basis of the SPADI.^6^ The acceptability of this model was further confirmed by evidence of the number of extracted factors in this study, using PA and the K-over-1 rule. All items were assigned to their respective factors as originally specified.^6^
**Model 3** tested two first-order correlated factors as indicated earlier, except that the results of EFA were used to assign disability items 6, 7, and 2 to the “Pain” factor.

### CFA method and interpretation

As data were ordinal and non-normal, the diagonally weighted least squares (DWLS) extraction method, based on polychoric correlations and asymptomatic covariances, was used.^20^ Relationships between items and factors were estimated using the DWLS method. The following fit indices were considered satisfactory: root mean square approximation <0.05 close fit, <0.08 good fit, <0.1 satisfactory fit; comparative fit index >0.95, and standard root mean square residual <0.08 good, <0.05 close fit.^30^

## Results

### Sample characteristics

The study population (n=660), comprised 413 (63.8%) males and 245 (37.2%) females (2 missing data) with a mean age of 63.1 years (standard deviation 7.4). Complete outcomes data were received from 648 (648/660, 98.2%) and 628 (628/660, 95.2%) participants for the OSS and SPADI questionnaires, respectively.

### EFA

#### OSS

The Kaiser–Meyer–Olkin (0.93), and Bartlett’s test of sphericity (*x*^2^ 5270.38; *P*<0.0001) values indicated that OSS data were highly suitable for FA.

Depending on the method employed, one- or two-factor models were suggested for the OSS. However, where a second factor was suggested, it appeared fairly weak (see Table 1; Figure S1 shows scree test results). Table 2 demonstrates the factor loadings based on the K-over-1 rule.

The EFA solution produced a main “Function” factor, containing 9 items, albeit with considerable cross-loading (eigenvalue > 0.3 on both factors; marked with bold text on Table 2) involving two of these items (item 11: “How much has pain from your shoulder interfered with your usual work (including housework)?”; item 2: “Have you had any trouble dressing yourself because of your shoulder?”). A second “Pain” factor (eigenvalue 1.23), contained just three items: 1 (“How would you describe the worst pain you had from your shoulder?”), 8 (“How would you describe the pain you usually had from your shoulder?”), and 12 (“Have you been troubled by pain from your shoulder in bed at night?”). These two factors together explained 67.7% of the variance (the second factor accounting for 10.3%) and were highly correlated (*r*=0.70). Cronbach’s alpha was 0.91 for all 12 OSS items and also for the 9-item Function factor, in each case, indicating a high degree of internal consistency; and 0.71 for the 3-item Pain factor, representing satisfactory (but suboptimal) internal consistency.

#### SPADI

Kaiser–Meyer–Olkin (0.94) and Bartlett’s test of sphericity (*x*^2^ 5639.56; *P*<0.0001) values indicated that the data were highly suitable for FA. As with the OSS, one- or two-factor models were suggested, depending on the test method employed (see Table 1; Figure S2 shows scree test results). Table 3 demonstrates the factor loadings based on the K-over-1 rule.

EFA produced a two-factor solution. A main “Pain” factor (eigenvalue 7.56), containing 8 items, including all 5 Pain items, as originally conceptualized, together with 3 items originally considered components of the Disability scale (Disability items 6: “Placing an object on a high shelf ”; 7: “Carrying a heavy object of 10 pounds”; 2: “Washing your back”). However 2 items exhibited considerable cross-loading (Pain scale item 4: “Touching the back of your neck” and Disability item 2: “When lying on the involved side”). The second “Disability” factor (eigenvalue 1.12) contained the remaining 5 Disability items, where 1 item cross-loaded (Disability item 3: “Putting on an undershirt or pullover sweater”). These two factors together explained 66.75% of the variance (the second factor accounting for 8.59%) and were highly correlated (*r*=0.76). Cronbach’s alpha was 0.94 for all 13 SPADI items; 0.91 for the 8-item “Pain factor” as here derived by EFA (0.87 for the 5-item Pain scale as originally conceptualized). The alpha was 0.90 for the 5-item “Disability factor” derived by EFA (0.93 for the original Disability scale containing all 8 Disability items).

### CFA

#### OSS

CFA (Table 4) indicated that both two-factor models of the OSS demonstrated marginally better fit than the one-factor model. However, neither one- nor two-factor models tested were rejected.

Results of EFA and CFA demonstrate that the OSS can be used both as a single summary score (as originally conceptualized) and in the form of Pain and Function component subscales/domains. Response scores from items 1, 8, 11, and 12, (each scored 0–4) can be summed into a “Pain” component and items 2–7,9, and 10 can be summed into a “Function” component. Cronbach’s alpha was 0.90 for this 8-item Function factor, indicating a high degree of internal consistency; and 0.75 for the 4-item Pain factor, representing satisfactory internal consistency.

#### SPADI

There was a marginally better fit for Model 2 (two factors, as the SPADI was originally conceptualized) (Table 5). As with the OSS, neither one- nor two-factor models were rejected.

## Discussion

The purpose of this study was to explore the underlying structure of the OSS and SPADI shoulder-specific PROMs in a large dataset (which increases confidence in findings), albeit within the context of a RCT with narrowly defined inclusion criteria and clinical characteristics (rotator cuff tear). The RCT’s diagnostic focus could potentially have consequences for the generalizability of our findings, since both PROMs were originally developed and validated as shoulder-specific measures appropriate for a wide range of shoulder problems and this would have brought about the original item content and measurement properties, and thus the results of any earlier validation work. Nonetheless, the majority of shoulder problems and surgery are concerned with disease or trauma affecting the rotator cuff; the RCT recruited from a wide range of centers nationally, had a large sample size, and obtained high questionnaire response rates.^9^ We are therefore reasonably confident that the findings of this paper are likely to be generalizable. Factor analytic techniques revealed the structure of these two widely used shoulder PROMs that should help to guide their practical application.

FA is one of a number of techniques that may be used to guide development of PROMs and their item content. Nonetheless, EFA and CFA are complex procedures with results influenced by the chosen analytic approach; none are definitive and care must be taken when generalizing beyond the study sample.^31^ Some widely used and respected PROMs have been developed without using this technique (eg, SF-36,^32^ EQ-5D^33^). Other considerations also apply in deciding the content and structure of a PROM. Since PROMs are intended to represent patients’ perspective, and be acceptable to patients (thereby encouraging high response rates), such considerations include the extent to which the instrument represents substantive salient aspects of the intended construct and equate with the measure’s content validity.^34^ Content validity is best assured using qualitative methods during questionnaire development, with patients’ insights obtained via interviews or focus groups informing item content and saliency, then cognitive debriefing to check received meaning and suitability of the wording. Evidence of other types of validity or reliability cannot overcome problems with content validity.^2^ In this regard, the interpretation of the results of the FA reported in this paper was considered in relation to the conceptualization and the content validity of the measures.

The OSS was conceptualized as a composite measure of Pain and Function, where, in developmental interviews, both aspects were frequently experienced as overlapping or inextricably linked. Different factor extraction methods indicated that the OSS can be understood as consisting of either one or two common factors. In a two-factor solution, however, the distinction between self-reported pain and function was somewhat indistinct (ie, the second factor was “weak” and some items cross-loaded), suggesting that shoulder pain and function are constructs that might indeed have some overlap, particularly in the way the patients perceive them, with pain influencing functional ability. While a Pain subscale consisting of either 3 or 4 items could nonetheless be supported, for researchers wishing to investigate pain separately, we would recommend including 4 items (items 1, 8, 11, and 12=Pain, all 8 remaining items=Function) as each asks about pain, and this model was associated with good internal inconsistency. We further recommend scoring each of the two-component subscales on a scale of 0 (worst) to 100 (best):

$$\text{Converting raw score to}\;0-100\;\text{scale}:\frac{100}{\text{Maximum possible domain score}}\times \text{Actual score}$$

While the SPADI was originally conceptualized as two separate (Pain and Disability) subscales, EFA produced findings that were similar to the analyses of the OSS: one or two common factors were supported and the distinction between self-reported pain and disability lacked clarity. EFA produced a solution at odds with the measure’s original conceptualization, although CFA confirmed that the original Pain and Disability item schema had marginally better fit than those suggested by the EFA. Nevertheless, both options were supported. Previous published results of EFA of the SPADI^6^,^7^,^35^ have used different study contexts (all observational, nonsurgical) and analytic approaches from the current study. However, in each case, where EFA identified the conceptualized “Pain” and “Disability” subscales, the demarcation between them was also found to be quite unclear. The different contexts, sample sizes, and techniques characterizing previous studies exploring the scale structure of the SPADI and most recently, a Persian version of the OSS^36^ (which used different analytic techniques and presented broadly similar findings) underline the importance of this study’s findings, where a direct comparison of these two measures was made possible within one large-scale study. The techniques used (based on recommended polychoric correlations) to surmise the optimal number of underlying domains, use of oblique rotation in EFA, and additional assessment of the fit of resulting hypothesized models represent a comprehensive and meticulous assessment, which gives confidence to the findings.

## Conclusion

The results of this study suggest that the OSS and SPADI Pain and Function/disability subscales each provide scope for additional analyses in the context of clinical trials or more generally. Further research is needed to calculate minimal change estimates, eg, minimum clinically important difference for each subscale. In particular, if used in trials that are specifically targeting either pain, or functional improvement, these subscales could be used as primary endpoints and, using a minimal change estimate, to calculate study power and sample size.

## Supplementary materials

Figure S1 Oxford shoulder score scree test.

Figure S2 Shoulder pain and disability index scree test.

## Figures and Tables

**Table 1:** Factor extraction results

Method	OSS (n=648)	SPADI (n=628)
Velicer’s MAP test	1	1
Horn’s PA	2	1
PA-MRFA	1	2
Scree test	2	1–2^b^
Kaiser’s eigenvalue-over-1 rule*	2^a^	2^b^

**Notes:** Suggested numbers of factors to be retained by the OSS and the SPADI, using different methods to assess dimensionality of the data (based on polychoric correlation matrices).

* Tables 2 and 3 for full details of exploratory factor analysis.

a Eigenvalue 1.233 for second factor.

b Scree test appears borderline between 1 and 2 factors. Eigenvalue 1.003 for second factor (see Supplementary materials).

**Abbreviations:** MAP, minimum average partial; OSS, Oxford shoulder score; PA, parallel analysis; PA-MRFA, PA based on minimum rank factor analysis; SPADI, shoulder pain and disability index.

**Table 2:** Exploratory factor analysis solution for the OSS

Pattern matrix^a^			

OSS questionnaire item number		Factor	
		1	2
6	OssTray1	0.972	−0.213
4	OssKnFk1	0.834	−0.040
9	OssHang1	0.777	0.031
7	OssBrHr1	0.767	−0.024
10	OssWash1	0.721	0.139
5	OssShop1	0.708	0.075
3	OssTran1	0.685	0.062
11	OssWork1	**0.493**	**0.362**
2	OssTrDr1	**0.456**	**0.440**
1	OssWsPn1	−0.082	0.836
8	OssUsPn1	0.054	0.766
12	OssPnNt1	−0.099	0.706

**Notes:** Extraction method: Principal axis factoring. Rotation method: Promax with Kaiser normalization. (Items that cross-load on both factors are shown in bold font.)

a Rotation converged in three iterations. Bold text denotes cross-loading.

**Abbreviation:** OSS, Oxford shoulder score.

**Table 3:** Exploratory factor analysis solutions for the SPADI

Pattern matrix^a^		

SPADI Questionnaire item number within customary scale	Factor	
	1 Pain	2 Disability
PainScale3	0.920	−0.081
PainScale1	0.801	−0.165
PainScale2	0.768	−0.091
DisabilityScale6	0.717	0.144
PainScale5	0.618	0.141
DisabilityScale7	0.531	0.264
PainScale4	**0.516**	**0.322**
DisabilityScale2	**0.475**	**0.387**
DisabilityScale5	−0.189	0.943
DisabilityScale4	−0.155	0.919
DisabilityScale1	0.255	0.608
DisabilityScale3	**0.305**	**0.553**
DisabilityScale8	0.259	0.496

**Notes:** Extraction method: Principal axis factoring. Rotation method: Promax with Kaiser normalization. Bold text denotes cross-loading.

a Rotation converged in three iterations.

**Abbreviation:** SPADI, shoulder pain and disability index.

**Table 4:** Summary of CFA fit measures for one- and two-factor models of the OSS

Model specification	*χ*^2^	*df*	CFI	SRMR	RMSEA	RMSEA 90% CI
**Model 1** All 12 items=single underlying factor	388.16	54	0.973	0.0710	0.080	(0.0706; 0.0892)
**Model 2** 2 factors: Pain=Items 1, 8, and 12 Function=all other items	277.32	53	0.982	0.0539	0.0726	(0.0633; 0.0823)
**Model 3** 2 factors: Pain=Items 1, 8,11 and 12 Function=all other items	331.00	53	0.978	0.0589	0.0772	(0.0679; 0.0868)
**Model 4** 2 factors: Pain=Items 1, 8,12, 11, and 2 Function=all other items	296.44	53	0.981	0.0564	0.0761	(0.0668; 0.0857)

**Abbreviations:** CFA, confirmatory factor analysis; CFI, comparative fit index; CI, confidence interval; *df*, degrees of freedom; OSS, Oxford shoulder score; RMSEA, root mean square approximation; SRMR, standard root mean square residual.

**Table 5:** Summary of CFA fit measures for one- and two-factor models for the SPADI

Model specification	*χ*^2^	*df*	CFI	SRMR	RMSEA	RMSEA 90% CI
**Model 1** All 13 items=single underlying factor	619.02	65	0.970	0.0058	0.0849	(0.0764; 0.0936)
**Model 2** 2 factors: Pain=Pain items 1–5 Disability=Disability items 1–8 – as originally specified	540.71	64	0.974	0.0500	0.0772	(0.0686; 0.0861)
**Model 3** 2 factors: Pain=Pain items 1–5 + Disability items 6, 7, and 2 Disability=all other Disability items	533.40	64	0.974	0.0498	0.0841	(0.0755; 0.0928)

**Abbreviations:** CFA, confirmatory factor analysis; CFI, comparative fit index; CI, confidence interval; *df*, degrees of freedom; RMSEA, root mean square approximation; SPADI, shoulder pain and disability index; SRMR, standard root mean square residual.
