# Supplementary figures and images for: A comparison of the Oxford shoulder score and shoulder pain and disability index: factor structure in the context of a large randomized controlled trial
Source: Patient Relat Outcome Meas. 2016 Nov 21;7:195–203. doi: 10.2147/PROM.S115488 (PMC5123655; doi:10.2147/PROM.S115488)

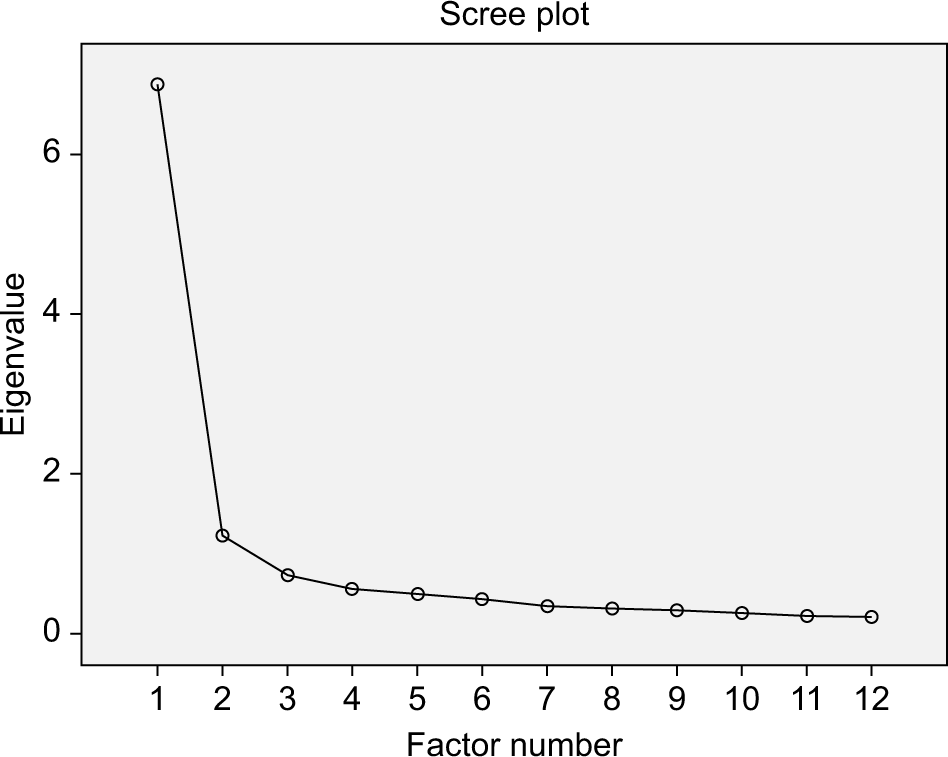

Supplement: Figure S1 — Oxford shoulder score scree test. [file prom-7-195s1.tif]

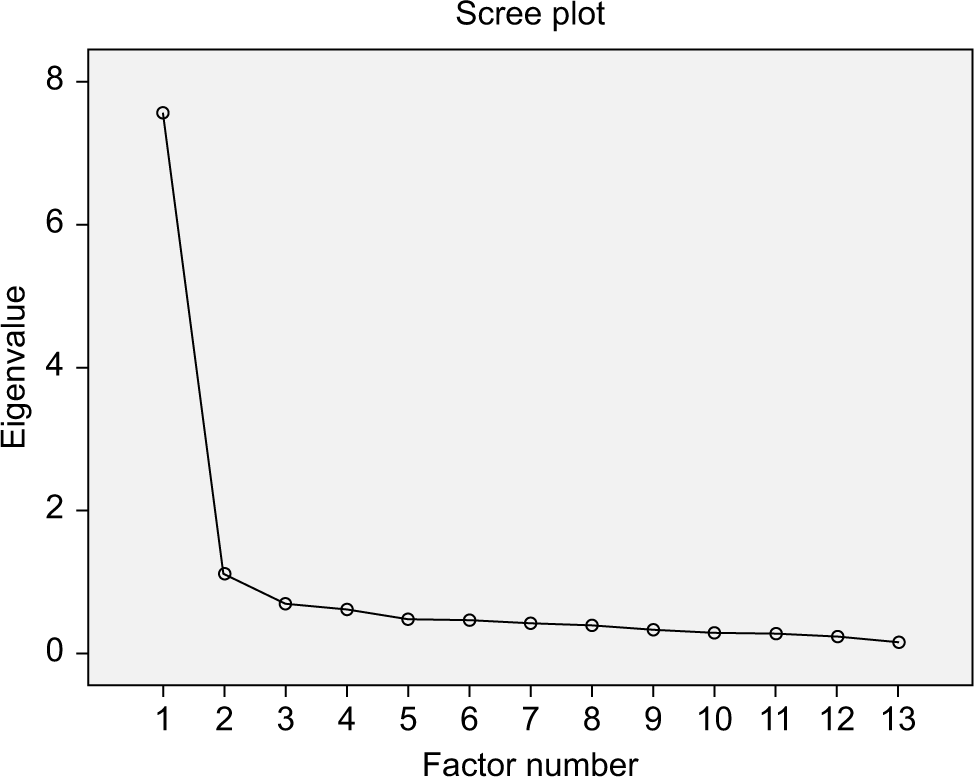

Supplement: Figure S2 — Shoulder pain and disability index scree test. [file prom-7-195s2.tif]
